# Novel Regulation of CCL2 Gene Expression by Murine LITAF and STAT6B

**DOI:** 10.1371/journal.pone.0025083

**Published:** 2011-09-28

**Authors:** Xiaoren Tang, Yu Yang, Salomon Amar

**Affiliations:** Center for Anti-inflammatory Therapeutics, School of Dental Medicine, Boston University, Boston, Massachusetts, United States of America; Università degli Studi di Milano, Italy

## Abstract

Inflammation is a multifaceted process: beneficial as a defense mechanism but also detrimental depending on its severity and duration. At the site of injury, inflammatory cells are activated by a cascade of mediators, one of which is LITAF, a transcription regulator known to upregulate TNF-α. We previously showed that human LITAF forms a complex with human STAT6B, which translocates into the nucleus to upregulate cytokine transcription. To dissect the molecular implications of this complex, a murine model was developed and interactions between mouse STAT6B (mSTAT6B) and mouse LITAF (mLITAF) were analyzed. Both mLITAF and mSTAT6B expression were MyD88- and TLR ligand-dependent. Furthermore, mLITAF was found to mediate LPS-induced CCL2 gene transcription with the cooperation of mSTAT6B leading to CCL2 protein expression. In LITAF-deficient mice, mLITAF-mediated CCL2 production in macrophages was significantly reduced compared to the wild-type control animals. Mice knockdown for mSTAT6B by 6BsiRNA1 tail vein injection resulted in a decrease in serum TNF-α and CCL2 production. mLITAF/mSTAT6B complex is proposed to play a role in LPS-induced CCL2 expression and possibly other cytokines.

## Introduction

The inflammatory response is the body's defense mechanism against harmful stimuli or damage. However, abnormal and prolonged inflammatory reactions can be detrimental and are related to immune system disorders [1], [2], atherosclerosis [3], [4], arthritis [5], [6], heart diseases [7], [8] and cancer [9], [10]. At the site of injury, a cascade of mediators, including cytokines (*e.g.* TNF-α, IL-1, IL-6 and CCL2), eicosanoids and reactive oxygen intermediates, activate nearby inflammatory cells such as macrophages and granulocytes, and promote additional leukocytes to extravagate from blood vessels and to infiltrate the surrounding tissues. CCL2, also known as monocyte chemoattractant protein-1 (MCP-1), is a chemokine associated with cerebral ischemia and rheumatoid arthritis [1], insulin resistance [11] and prostate cancer [2]. As cytokine or chemokine dysregulation has been implicated in inflammatory diseases, it is important to establish the manner in which chemokine or cytokine gene expression is controlled, thus providing potential therapeutic intervention in these diseases [3].

It is known that transcription factors must translocate into the nucleus using a nuclear core complex (NPC) to achieve gene transcription. We previously cloned and characterized a transcription factor that we named LPS-Induced TNF-Alpha Factor (LITAF) [12]. The cytoplasm-nucleus shuttling of LITAF sequentially enhances the transcription of the tumor necrosis factor-alpha (TNF-α) [13]. Proteins like LITAF contain a nuclear localization signal(s) (NLS) that facilitates translocation into the nucleus [14].

The present study confirms that just like in human [13], [15], both mouse LITAF (mLITAF) and mouse STAT6B (mSTAT6B) expression were MyD88- and TLR ligand-dependent. In addition LPS-induced mSTAT6B directly interacts with mLITAF and forms a complex in cytoplasm. This complex then translocates into the nucleus, where it upregulates the transcription of several inflammatory cytokines. Finally, p38α MAP kinase was found to mediate mLITAF gene expression and inhibition of p38α blocked LITAF nuclear translocation.

Building on these data, we further demonstrate that the knockdown of mSTAT6B by 6BsiRNA1 prevented mLITAF from regulating CCL2 production but mSTAT6B does not play a role in regulation of CCL2 production in the absence of mLITAF. Tail vein injection of 6BsiRNA1 in mice resulted in the reduction of LPS-induced serum TNF-α or CCL2 levels in mice. The present data implicate mLITAF/mSTAT6B complex in LPS-induced TNF-α and CCL2 expression and discuses the potential role of this complex in other cytokine gene expression for the control of inflammatory processes.

## Materials and Methods

### Animals, cell lines, and bacteria

Wild-type and genotype deficient C57BL-6 mice (TLR-2^−/−^ Cat# 004650, TLR-4^−/−^ Cat# 007227-9, or MyD88^−/−^ Cat#009088) were purchased from The Jackson Laboratory. The mLITAF conditional knockout mouse strain (macLITAF^−/−^) was generated by our laboratory as described previously [15]. All mice protocols used in this study have been approved by Boston University Animal Care and Use Committee. Mouse peritoneal macrophages were elutriated from wild type and genotype deficient mice (LITIF^−/−^), and purified by conventional methods as described [4]. RAW264.7 murine macrophage cells were supplied by ATCC. All bacterial cloning constructs used were *E. coli* strain DH5α (Invitrogen, Carlsbad, CA). Strain 381 of *P. gingivalis* was grown in brain heart infusion broth with hemin (5 µg/ml) and menadione (1 µg/ml) in an anaerobic atmosphere (85% N_2_/10% H_2_/5% CO_2_) for 24–48 h at 37°C before preparation of LPS as we have described [16].

### Mice and tail vein injection

Age and weight-matched wild-type mice (C57BL/6) were selected and treated with tail vein injection [5] with *E.coli* LPS and/or RNAi (Table 1). Following the injection, serum TNF-α concentration from each mouse was measured by ELISA and mice were monitored for up to 72 hrs.

**Table 1:** Tail vein injection of *E.coli* LPS plus 6BsiRNA1 or 6BsiRNA2 as the control.

No. of group (6mice/group)	Tail vein injection				
	Untreated	1 mg LPS/mouse	40 uM m6BsiRNA2/mouse	20 uM m6BsiRNA1/mouse	40 uM m6BsiRNA1/mouse
1	+				
2			+		
3					+
4		+			
5		+	+		
6		+		+	
7		+			+

### Design of siRNAs

Two siRNA sequences, 6BsiRNA1: 5′-GATGTCACTCCCTATTTCATA-3′ for silencing mSTAT6B gene expression, and 6BsiRNA2: 5′-GAGCACTCCATGGCTGTCTTT-3′ as the negative control were designed by a commercial software (www.sirnawizard.com/design_advanced.php). Their RNA sense strand and antisense strand were synthesized (Qiagen) and used for promoter assay and ELISA as described below.

### Plasmid DNA constructs

#### (1)

The mouse LITAF DNA (mLITAF) fragment subcloned into the pCDNA3HA [13] vector were obtained by our laboratory.

#### (2)

The mouse mSTAT6B in-frame DNA fragment (bankit1369793; submitted to GenBank) was generated by PCR reaction with primer pairs: 5′-ATGGCCCGAC GGAACCCTTC-3′ and 5′-GTCATCTTGATGGTAGCT-3′ and subcloned into the pcDNA3HA and named mSTAT6B.

#### (3)

The mouse CCL2 5′UTR&promoter (Genebank Access# NT-096135, −1034 to +1 bp relative to the ATG start codon) sequences were selected. Five DNA constructs with CCL2 5′UTR&promoter sequential deletions were generated and amplified by PCR with a same reverse primer: 5′-TGGTGGTGGAGGAAGAGA-3′ and five diversified forward primers: 1) 5′-ACGAAGGAAACAGGGCAGAG-3′ for mCL2Pwt (−1034∼+1 bps), 2) 5′-TGACCCTTCT GGACCTCAGC-3′ for mCL2P840 (−840∼+1 bps), 3) 5′-TATTCAACAAGGCCTGATAA-3′ for mCL2P560 (−560∼+1 bps), 4) 5′-TTCCTTTTATTTCAGTGAAA-3′ for mCL2P420 (−420∼+1 bps), and 5) 5′-AAAATACCAAATTCCAACCC-3′ for mCL2P280 (−280∼+1 bps). The individual PCR products of CCL2-5′UTR&promoter (1034 bps, 840 bps, 560 bps, 420 bps and 280 bps) were subcloned into the luciferase reporter vector, pGL3-basic.

#### (4)

The DNA fragments of 5′UTR&Promoter of CCL1(AL713839), IL-1β( NT_039207.7), IL-6 (NT_162294.3), and IL-10(HQ014592) were generated by PCR with mouse genomic DNA (obtained from our lab) as template and primer pairs 5′-tagacagacaggatcaacct-3′&5′-ggtgataagtttctgtctca-3′, 5′-aagtgcgtgtctctccagaa-3′&5′-agctgcttcagacacctg-3′, 5′-ctccttgcatgacctggaaa-3′&5′-agcggtttctggaattgact-3, and 5′-cttgcccagggtacagaa-3′&5′-gatggagctctcttttct-3′. The individual PCR products of 5′UTR&promoter (named CCL1-p, IL-1β-p, IL-6-p, or IL-10-p) were subcloned into the luciferase reporter vector, pGL3-basic.

### Preparation of protein extracts and Western blot analysis

The cell lysate from mouse primary macrophages were subjected to SDS-PAGE and transferred to a membrane then blotted with antibodies against mouse mSTAT6B (5278, BioSynthesis, Inc), LITAF (Cat #611614, BD Biosciences), NF-kB p65 (RelA, sc-372), CSF-1 (LS-C104794-50, BioSciences) or Actin as control for Western blot analysis.

### ELISA

The conditioned media from Mouse Raw 264.7 cells or mouse primary macrophages or plasma samples from mice were subjected to ELISA for detection of endogenous expression of MCP-1 (CCL2, Invitrogen), TNF-α (Invitrogen), MCP-2 (biocompare), IL-1α (Invitrogen), IL-1β (Invitrogen), IL-6 (Invitrogen), IL-10 (Invitrogen), or RANTES (Invitrogen). Total protein concentration of corresponding cell lysate was measured and used for normalization.

### Luciferase assay

Mouse Raw 264.7 (1×10^6^) cells were transiently transfected/cotransfected with appropriate promoter-reporter DNA alone such as mCL2Pwt, mCL2P840, mCL2P560, mCL2P420, and mCL2P280, and/or plus mSTAT6B, mLITAF, both mSTAT6B/mLITAF or pcDNA3 as control by LipofectAMINE 2000 (Invitrogen Cat#11668019). The luciferase activity from the treated cells was quantified with a Turner Designs luminometer model TD-20/20 by reaction of the appropriate transfected cell lysate 1∶1 (vol/vol) with the substrate solution (Cat#E151A, Promega). Triplicate assays were performed and the data was analyzed. Values were normalized to β-gal production and graphed.

### Immunoprecipitation

RAW264.7 murine macrophages grown in RPMI medium 1640 with 10% FBS were transfected with mLITAF or mSTAT6B for 24 h. Cells were rinsed once with ice-cold PBS and routinely lysed in the lysis buffer (Promega) supplemented with protease inhibitors. Lysates were precleared by pre-incubation with protein A/G-agarose-Sepharose beads (Cat #sc-2003, Santa Cruz Biotechnology) for 30 min, and incubated at 4°C for 2 h with 2 µg of HA antibody, followed by addition of 20 µl of protein A/G plus-agarose-Sepharose beads for another hour. The beads were washed three times in PBS then the immune complexes were released from the beads by boiling in SDS sample buffer for 5 min. Immunoprecipitated products were analyzed by EMSA.

### Chromatin Immunoprecipitation (ChIP)

Experiments were performed using a commercial kit (Cat#: 53009, ChIP-IT Express Enzymatic, Active Motif) with some modifications. 1×10^6^ Raw 264.7 cells were transfected with 1 µg of mLITAF DNA or 1 µg of mSTAT6B DNA overnight. The 10 µg nuclear extracts (NE) as input from the cross-linked cells were immunoprecipitated (IP) with 1 µg LITAF antibody, 1 µg 5278 (anti-mSTAT6B) antibody or 1 µg normal IgG as control at 4°C for 4 hrs. DNAs from each experimental group (input or IP) were isolated by elution, reverse cross-linking and Proteinase K treatment according to the manufacturer's instruction. The DNA was then used as template to perform PCR with primer pairs (A+B, C+D, E+F, G+H, G+I, or I+J for amplification of DNA fragment of CCL2 5′UTR&promoter by PCR. GAPDH primer pairs (Invitrogen) as control was used at the same condition.

### EMSA

A commercial kit, Gel Shift Assay System (Promega), was used. A reaction mixture contained 2 µL of HeLa nuclear extract, 1×10^5^ cpm/µL radiolabeled double-stranded oligo DNA probe, an appropriate concentration of *E. coli* protein extraction, HA-Immunoprecipitated protein (IP-mLITAF or IP-mSTAT6B), 2 µL of 5× reaction buffer, and nuclease-free water to achieve a final volume of 10 µL. Mixtures were incubated at room temperature for 20 min, followed by electrophoresis on nondenaturing 6% polyacrylamide gels in Tris-borate-EDTA buffer (90 mM Tris-borate, 2 mM EDTA and HEPES, at pH 8.0). The gel imaging was developed.

### Statistical analysis

The SAS software package was used for all statistical analyses. All experiments were performed at least in triplicate. All the data were normally distributed. In case of multiple mean comparisons, data were analyzed by analysis of variance (ANOVA). In case of single mean comparison, data were analyzed by Student's t-test. In case of time-course study, data were analyzed by two-way repeated measure ANOVA, and P values less than 0.05 were regarded as significant.

## Results

### TLR involvement in mLITAF/mSTAT6B signaling pathway

**Figure 1:**
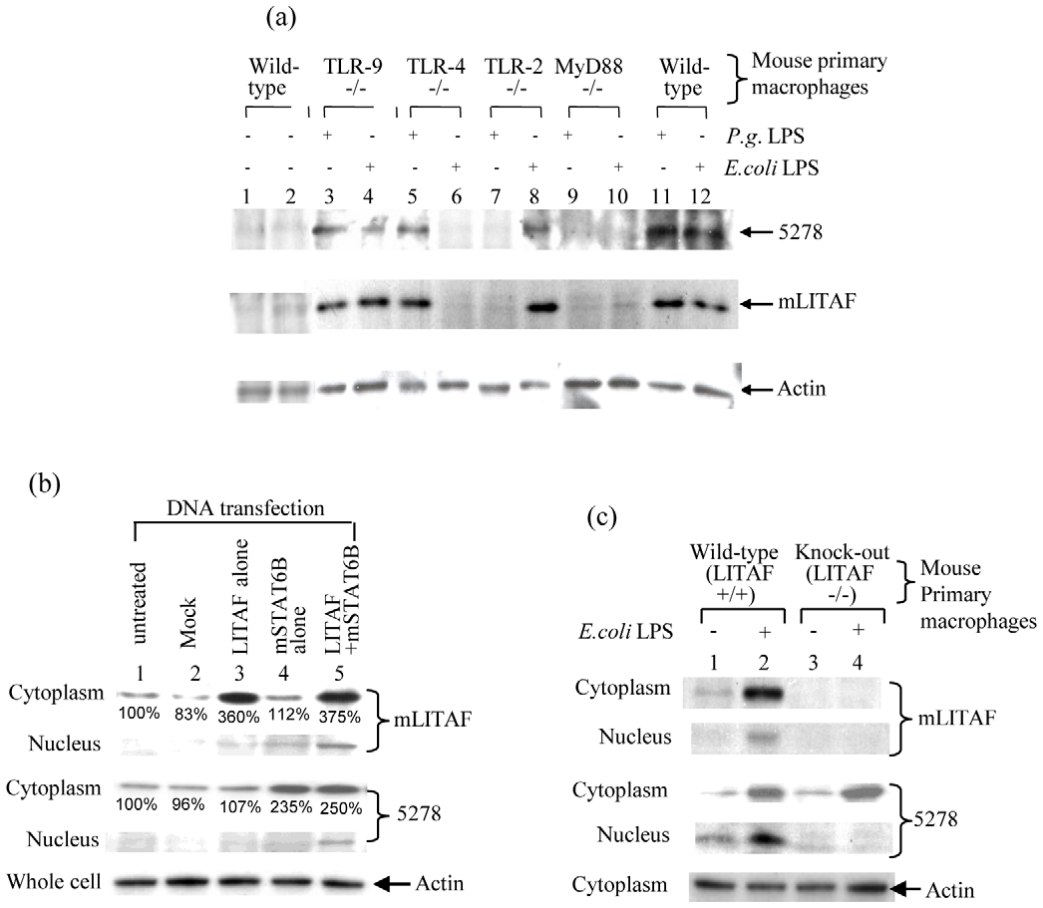
mSTAT6B/mLITAF signaling pathway. (a) The primary macrophages collected from various deficient genotype mice (No. 1&2 and 11&12 for wild-type as the control, No. 3&4 for TLR-9^−/−^, No. 5&6 for TLR-4^−/−^, No. 7&8 for TLR-2^−/−^, or No. 9&10 for MyD88^−/−^) were treated with 0.1 µg/ml *P. gingivalis* LPS (No. 3,5,7,9&11) or 0.1 µg/ml *E. coli* LPS (No. 4,6,8,10&12) for 2 hrs and washed with PBS and cultured overnight. The cell lysate from each test group was subjected to Western blot with antibodies against mSTAT6B (5278), mLITAF or Actin as the control. The assays were performed in triplicate and a representative experiment is presented. (b) Mouse macrophages were transfected with either mLITAF alone or mSTAT6B alone or cotransfected with both mLITAF and mSTAT6B. Proteins from cytoplasm and nucleus of cells were purified separately and subjected to Western blot analysis using antibodies against mLITAF, mSTAT6B (5287), or Actin (control) as indicated by arrows. The assays were performed in triplicate and a representative experiment is presented. The protein band intensity was analyzed using VersaDoc Imaging System model 4000 MP with Quantity One Quantitation Software version 4.6.3 (Bio-Rad). The band intensity from cytoplasm of untreated cells was used as control and assigned 100%. The other bands were evaluated compared to the control and their intensities with % were attached. (c) Mouse macrophages collected from wild-type mouse (No. 1&2) or from LITAF-deficient genotype mice (No. 3&4) were treated with 0.1 µg/ml *E.coli* LPS (No. 2&4) or untreated as control (No. 1&3) for 2 hrs and washed with PBS and cultured overnight. Proteins from cytoplasm and nucleus of treated cells were purified separately and subjected to Western blot with the antibodies against mLITAF, mSTAT6B (5287), or Actin as control (indicated by arrows). The assays were performed in triplicate and the representative experiment a shown.

To determine TLR engagement in mLITAF/mSTAT6B signaling, mLITAF or mSTAT6B protein levels in response to LPS stimulation in mouse macrophages of various genotypes (TLR-2^−/−^, -4^−/−^, -9^−/−^, MyD88^−/−^, and wildtype as control) were measured by Western blot. As shown in Fig. 1a, both mSTAT6B and mLITAF were induced by either *P. gingivalis* or *E. coli* LPS in TLR-9^−/−^ macrophages (No. 3&4) but not induced at all in MyD88^−/−^ macrophages (No. 9&10). This implied that TLR-9 was not involved in LPS-signaling pathway, while MyD88 is required for signal transmission and thus the pathways are myD88 dependent. In TLR-4^−/−^ macrophages, mSTAT6B and mLITAF were induced by *P. gingivalis* LPS (TLR-2 ligand) (No. 5) but not by *E. coli* LPS (TLR-4 ligand) (No. 6), while in TLR-2^−/−^ macrophages, mSTAT6B and mLITAF were induced by *E. coli* LPS (No. 8) but not by *P. gingivalis* LPS (No. 7) compared to control. These findings indicate that mLITAF and mSTAT6B induction occurred with either TLR-2 or TLR-4 engagement.

### Nuclear translocation of mLITAF with mSTAT6B as a complex

Analysis of the mSTAT6B sequence discloses only partial homology to human STAT6B and no homology to human STAT6 advocating for the existence of 3 distinct genes (See Information S1 and Figure S1). Either *E.coli* or *P.g.* LPS are capable of inducing mSTAT6B independently from mLITAF (See Information S1, and Figure S2). The pull-down assay showed that mLITAF associates with mSTAT6B via protein-protein interaction in response to LPS (See Information S1 and Figure S2b). Finally, phosphorylation of p38 kinase was involved in LPS/TLR2-4/mLITAF/mSTAT6B signaling pathway (See Information S1 and Figure S3) similar to human LITAF/STAT6B-dependent signaling pathway [13], [15].

To dissect the mechanism associated with the mLITAF/mSTAT6B complex formation and its nuclear translocation, mouse macrophage were transfected with mLITAF and/or mSTAT6B (Fig. 1b). Overexpression of either mLITAF alone (Fig. 1b, No. 3) or mSTAT6B alone (No. 4) did not yield a protein band in the nuclear lysate as compared to controls (No. 1&2). However, overexpression of both mLITAF and mSTAT6B (No. 5) produced a detectable band in the nucleus, indicating that nuclear translocation of these proteins were dependent on the formation of mLITAF/mSTAT6B complex. To further determine the dependence between mLITAF and mSTAT6B in macrophages for their nuclear translocation in response to LPS stimulation, the nuclear or cytoplasmic lysate from *E. coli* LPS-treated mouse macrophages of wild-type (Fig. 1c, No. 1&2) or LITAF knock-out (No. 3&4) mice were detected by LITAF or 5278. As shown in Fig. 1c, neither mLITAF nor mSTAT6B protein was observed in the nuclear lysate (No. 4) even though a good protein yield of mSTAT6B remained in the cytoplasm (No. 4) compared with controls. This suggests that mSTAT6B is LITAF-dependent and not able to translocate into nucleus in the absence of LITAF.

### The role of mLITAF/mSTAT6B in regulation of CCL2 gene expression

**Figure 2:**
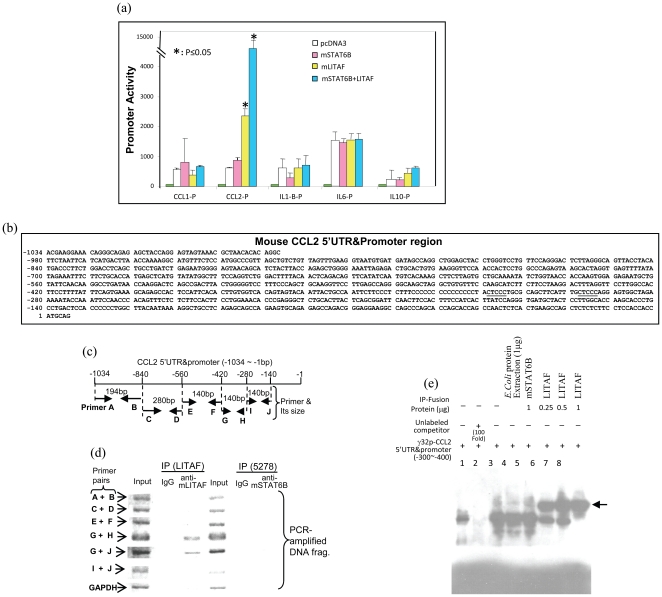
mSTAT6B and/or mLITAF-mediated cytokine promoter activities. (a) Raw264.7 cells were untreated as control (green bars) and transiently transfected with reporter genes of CCL1-p, CCL2-p, IL-1β-p, IL-6, or IL-10-p with the addition of pcDNA3 (white bars), mSTAT6B (pink bars), mLITAF (yellow bars), or both mSTAT6B and mLITAF (blue bars). All test cells were cotransfected with 5 ng of β-gal DNA. Cell lysate was then purified and examined by luciferase assay. Triplicate assays were performed and their values were normalized to β-gal production and graphed. (b) Diagram of mouse CCL2 5′UTR&promoter DNA sequence in the region from −1,034 to 1. The putative mLITAF binding site (CTCCC) in the region (−319∼−315, −298∼−294) is underlined. (c) Diagram of primer (A–J) location in mouse CCL2 5′UTR&promoter DNA and their size. (d) CHIP assay. DNAs from input group as control or CHIP group (LITAF or 5279) were used for PCR with primer pairs of A&B, C&D, E&F, G&H, G&J, or I&J. The control (input) group showed DNA fragments that were amplified by PCR with all primer pairs. Fragments amplified by PCR with primer pairs G&H or G&J were shown only in the LITAF CHIP group. None of the PCR-amplified DNA fragments in mSTAT6B (5278) CHIP group were observed. The assays were performed in triplicate and a representative experiment is presented. (e) EMSA. HA-immunoprecipitated protein (IP) from each test cells was purified and added to the appropriate reaction buffer with the 32p-ATP-labeled double stranded CCL2 oligonucleotide (GCAGAGCCACTCCATTCACACTTGTGGTCACAGTAGTACAAT TACTGCCAATTCTTCCCTCTTTCCCCCCCCCCCCCCCTACTCCCTGCGCAGCTTCATTT) as DNA probe. Treatment with DNA probe alone in No. 1 and 3, with 100-fold excess of unlabeled competitor (No. 2), or plus 1 µg of *E. coli* extraction as control (No. 4), 1 µg IP-mSTAT6B (No. 5), different concentration of IP-mLITAF (0.25 µg in No. 6, 0.5 µg in No. 7, 1 µg in No. 8) were assessed by EMSA. The shifted bands are indicated by arrow. The assays were performed in triplicate and a representative experiment is presented.

To examine the biological function of mLITAF/mSTAT6B complex, a promoter assay was performed. As shown in Fig. 2a, overexpression of both mLITAF and mSTAT6B significantly increased CCL2 promoter activity. To define whether there is a consensus sequence for the binding of mLITAF to cytokine promoters, CCL2 and other cytokines. In order to determine the binding activity of mLITAF and mSTAT6B to CCL2 5′UTR&promoter, PCR primer pairs (A–J) were designed covering the CCL2 5′UTR&promoter region (Fig. 2b&c) and chromatin immunoprecipitation (ChIP) was further performed. As shown in Fig. 2d, the DNA fragment of CCL2 5′UTR&promoter was amplified by PCR with IP-mLITAF and primer pairs (G&H or G&J) but not detected with primer pairs (A&B, C&D, E&F or I&J). This suggests that the region from −420 to −280 of CCL2 5′UTR&promoter contains a binding site for LITAF interaction. Additionally, none of PCR-amplified DNA fragments were found with all primer pairs for IP-mSTAT6B, indicating that mSTAT6B does not directly interact with CCL2 5′UTR&promoter. The binding site of −420 to −280 was further confirmed by EMSA. As shown in Fig. 2e, the [^32^P]ATP-labeled double-stranded nucleotide −400 to −300 of CCL2 5′UTR&promoter was treated with IP-mSTAT6B or IP-mLITAF fusion proteins or E. coli protein extraction as control. The shifted DNA bands as indicated by arrows were observed with IP-mLITAF (Fig. 2e, No. 6-8) but not IP-mSTAT6B (No. 5) compared to the control (No. 4). These findings suggest that mLITAF functions as a binding factor in the regulation of CCL2 gene expression.

To determine whether the CTCCC sequence identified for the binding of mLITAF on TNF promoter was present in CCL2 as well as other mediators, bioinformatics analysis of the promoter sequences for some inflammatory mediators was performed. We found that TNF-α, CCL2 (MCP-1), MCP-2, IL-1α, IL-10, and RANTES which promoter contained a “CTCCC” sequence responded to LITAF-mediated stimulation. Consistent with this finding, cytokine promoters lacking a CTCCC sequence upstream of the transcription initiation site, such as IL-6 or IL-1β were not affected by LITAF (Table 2).

**Table 2:** 

Mediator	ELISA analysis of gene secretion induced by mLITAF overexpression (+/−)*	BLAST SEARCH of the location for the putative binding sequence, CTCCC.**
TNF-α	+	−16_∼_−20, −85_∼_−89, −155_∼_−159, −633_∼_−637
MCP-1	+	319_∼_−315, −298_∼_−294
MCP-2	+	−85_∼_−89
IL-1α	+	340_∼_−344
IL-10	+	−743_∼_−747, −867_∼_−871
RANTES	+	−22_∼_−26, −90_∼_−94, −138_∼_−142, −362_∼_−366
IL-6	−	None
IL-1β	−	None

*Raw264.7 cells were transfected with mLITAF or pcDNA alone as control o/n. The secretion protein in the conditioned medium was assessed by ELISA. 200% increase or more in protein concentration compared to the control was assigned with (+), but it was considered a minus (−) if the protein level was 100% or less than the control.

**Bioinformatic analysis of binding site of TNF-α (U68414), MCP-1 (AL713839), MCP-2 (AL713839), IL-1α (NT_039207.7), IL-10 (HQ014592), RANTES (NT_096135.5), IL-6 (NT_162294.3), or IL-1β (NT_039207.7) in their promoter region from −1 to −900.

**Figure 3:**
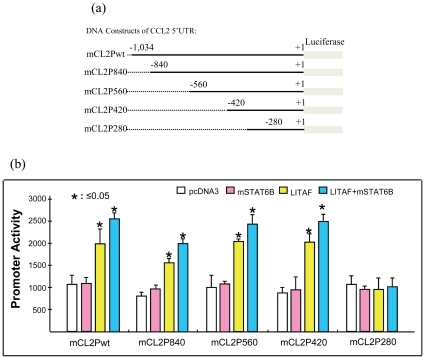
Effects of mSTAT6B and/or mLITAF on mouse CCL2 5′UTR&promoter-enhanced activity. (a) Diagram of DNA deletions of mouse CCL2 5′UTR&promoter region. The PCR-amplified DNA fragment of mouse CCL2 5′UTR&promoter in the region from −1034∼1, −840∼1, −560∼1, −420∼1, or −280∼1 was respectively inserted into pGL3-basic plasmid as reporter DNAs (named mCL2Pwt, mCL2P840, mCL2P560, mCL2P420, or mCL1P280). (b) Raw264.7 cells were cotransfected with reporter DNAs of mCL2Pwt, mCL2P840, mCL2P560, mCL2P420 or mCL2P280 plus pcDNA3 as control (white bars), mLITAF (pink bars), mSTAT6B (yellow bars) or both mLITAF&mSTAT6B (blue bars) overnight. All test cells were cotransfected with 5 ng of β-gal DNA. The lysate from each test cells was assessed by luciferase assay. Triplicate assays were performed and their values were normalized according to β-gal production and graphed.

**Figure 4:**
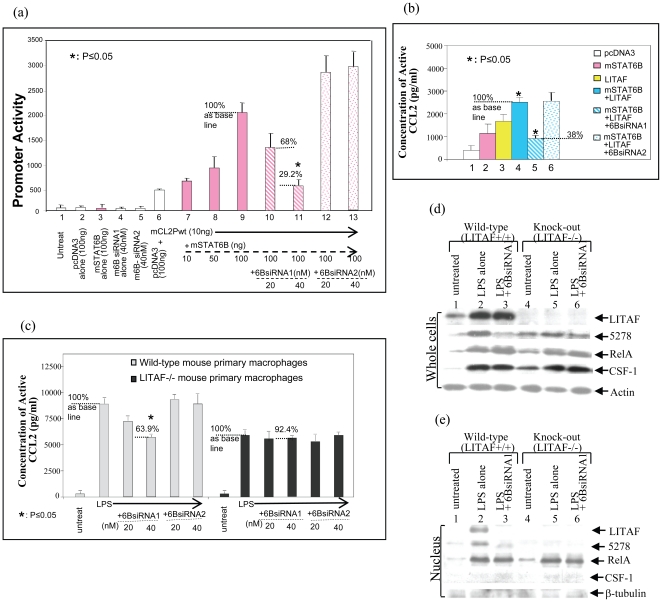
Downregulation of CCL2 gene transcription by 6BsiRNA1 silencing of mSTAT6B. (a) Downregulation of CCL2 promoter activity by 6BsiRNA1. Raw264.7 cells were untreated (No. 1) or transfected with pcDNA3 alone as control (No. 2), mSTAT6B DNA alone (No. 3), 40 nM m6BsiRNA1 alone (No. 4), 40 nM m6BsiRNA2 alone (No. 5), or cotransfected with mCL2Pwt (No. 6-13) plus pcDNA3 (No. 6), or plus different concentration of mSTAT6B DNA (No. 7-13). All experimental groups were cotranfsected with 5 ng of β-gal DNA. Treated cells were immediately added with 6BsiRNA1 (No. 4, 10&11) or 6BsiRNA2 as the negative control (No. 5, 12&13) and cultured overnight. The cell lysate from each test group was used for luciferase assay. Triplicate assays were performed and their values were normalized according to β-gal production and graphed. (b) Regulation of CCL2 gene expression by 6BsiRNA1. Raw264.7 cells were transfected with 100 ng pcDNA3 alone as control (white bars), 100 ng mSTAT6B DNA alone (pink bars), 100 ng mLITAF DNA alone (yellow bars) or cotransfection with both 100 ng mLITAF and 100 ng mSTAT6B DNAs (blue bars) plus 40 nM 6BsiRNA1 (striped blue bars) or plus 40 nM 6BsiRNA2 as control (dotted blue bars). All test cells were cotransfected with 5 ng of β-gal DNA. Treated cells were cultured overnight. The conditioned medium from each test group was used to detect CCL2 production by ELISA. Triplicate assays were performed and their values were normalized according to β-gal production and graphed. (c) Wild-type or LITAF^−/−^ mouse primary macrophages were untreated as control or treated with 100 ng/ml of *E. coli* LPS for 2 hrs and washed with PBS. The treated cells were added with different concentrations (20 or 40 nM) of 6BsiRNA1 or 6BsiRNA2 as negative control and cultured overnight. The conditioned medium from each test group was used to detect CCL2 production by ELISA and the protein concentration of the corresponding cell lysate was measured for normalization. Triplicate assays were performed and graphed. (d&e) Mouse macrophages collected from wild-type mouse (No. 1-3) or from LITAF-deficient genotype mice (No. 4-6) were treated with 0.1 µg/ml *E. coli* LPS (No. 2,3,5&6) or untreated as control (No. 1&4) for 2 hrs and washed with PBS. The treated cells were cultured overnight. Proteins from whole cells (panel d) or nuclei (panel e) were purified and subjected to Western blot with the antibodies against mLITAF, mSTAT6B (5287), RelA, CSF-1, or Actin as control (indicated by arrows). The assays were performed in triplicate and a representative experiment is presented.

To determine whether CCL2 promoter activity is dependent on mLITAF binding activity alone, CCL2 5′UTR&promoter reporter DNA deletions were constructed (Fig. 3a). This approach allowed us to also determine the location of the biding activity. As shown in Fig. 3b, transfection of mLITAF alone (yellow bars) or both mLITAF&mSTAT6B (blue bars) caused between 2 to 2.5 fold upregulation of CCL2 promoter activity while transfection of mSTAT6B alone (pink bar) did not have any activity promoter. This upregulated activity was maintained even until the 5′UTR&promoter region lacked the 5′UTR&promoter region from −1,134 to −420 and was lost when the region lacked from −1,134 to −280 suggesting that the critical binding activity was located between −420 and −280 5′UTR&promoter sequence. Furthermore, a dose course promoter assay was performed to show that an increasing concentration of mSTAT6B enhanced CCL2 promoter activity (Fig. 4a, No. 7-9). Consequently, knockdown of mSTAT6B using 6BsiRNA1 reduced CCL2 promoter activity to 68% (No. 10) and 29.2% (No. 11) in comparison to the effect of 100 ng of mSTAT6B alone as the 100% baseline (No. 9), while no significant reduction was observed with control 6BsiRNA2 (No. 12&13). To confirm this regulation at the protein level, Raw264.7 cells were transfected with mLITAF and/or mSTAT6B alone, or cotransfected with LITAF/mSTAT6B plus 6BsiRNA1 or 6BsiRNA2 as control. As shown in Fig. 4b, transfection with mLITAF/mSTAT6B caused a high CCL2 production in Raw264.7 cells, while knockdown of mSTAT6B by 6BsiRNA1 reduced CCL2 protein level to 38% (No. 5) in comparison to uninhibited mLITAF/mSTAT6B as the 100% baseline (No. 4). No significant reduction was observed with control 6BsiRNA2 (No. 6). Finally, in order to examine how endogenous mLITAF/mSTAT6B regulates CCL2 production, wild type or LITAF^−/−^ mouse primary macrophages were treated with 6BsiRNA1 and 6BsiRNA2 as control after LPS stimulation. The conditioned medium from each test group was assessed by ELISA. As shown in Fig. 4c, treatment with 40 nM of 6BsiRNA1 reduced LPS-induced CCL2 production to 63.9% compared to LPS alone as the 100% baseline in wild-type cells. However, no effect was observed under the same condition in LITAF^−/−^ cells. These results suggested that 1) knock down of mSTAT6B prevented mLITAF from regulating CCL2 production and 2) mSTAT6B did not play a role in regulation of CCL2 production when mLITAF was knocked out.

In addition, as it is well known that CCL2 gene expression in macrophages is upregulated by other factors such as RelA [17], [18] or CSF-1 [19] in response to LPS. Our investigation focused on whether cell transfection of 6BsiRNA1 would affect these factors as detected by Western blot anlaysis. As shown in Fig. 4d, RelA or CSF-1 production was induced by LPS in either wild-type (No. 2) or LITAF-deficient cells (No. 5). These LPS-induced proteins were not significantly reduced in the presence of 6BsiRNA1 (No. 3&6), whereas mSTAT6B production (5278) was reduced at the same condition (No. 3 or 6). However, RelA or CSF-1 protein translocation to nucleus was not affected by either mSTAT6B gene expression (No. 2) or gene knocked down (No. 3) in response to LPS (Fig. 4e).

**Figure 5:**
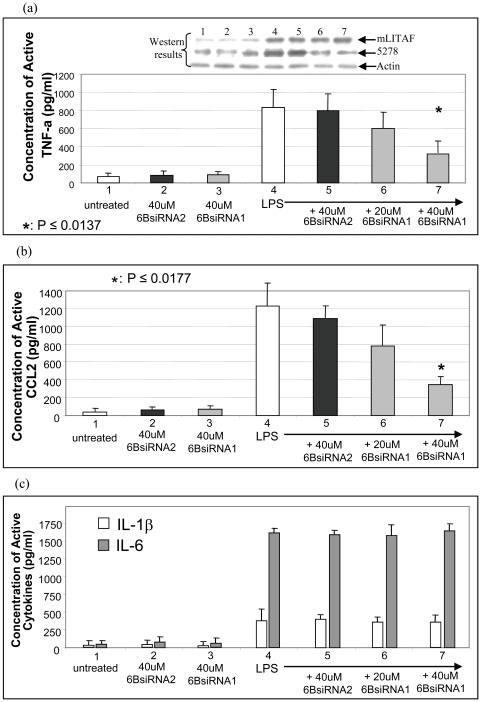
Effect of 6BsiRNA1 on LPS-induced serum TNF-α or CCL2 production. Mice (n = 6×6 groups) were treated as shown in Table 1. Total of 100 ul PBS solution containing 1 mg *E. coli* LPS plus 40 nM 6BsiRNA2 as control, or plus 20 nM 6BsiRNA1 or 40 nM 6BsiRNA1 was injected into each mouse. Blood was collected 5 hrs after injection. Mice were warmed under heating lamps to promote blood flow, and a small incision was made on the tail. About 50 µl of blood was collected per mouse. Red blood cells were removed from the sample via centrifugation at 5,000 rpm for 1 min using serum separator tubes. Top Panel: protein expression from each sample (Nos 1-7) detected by Western blot using antibodies against mLITAF, 5278 or Actin (control). Lower panel: serum levels of TNF-α (a), CCL2 (b) or IL-1β, IL-6 (c) from each sample measured by ELISA. ELISA immunoreactivity was quantified using a microplate reader and measurements were graphed. The assays were performed in triplicate and a representative experiment is presented.

Our previous study showed that LITAF-deficient macrophages reduced LPS-induced TNF-α production [13], [15] and data in this study indicate that knockdown of mSTAT6B by 6BsiRNA1 in macrophages decreased LPS-induced CCL2 production. Therefore we were interested to determine whether i.v. injection of 6BsiRNA1 in mouse protects animal from LPS-induced TNFα/CCL2 production. To address this, mice were treated as described (Table 1) by tail vein injection with one of the following: PBS alone (untreated, No. 1), 40 uM m6BsiRNA2 alone (No. 2), 40 uM m6BsiRNA1 alone (No. 3) or LPS (No. 4) plus 40 uM m6BsiRNA2 (No. 5) or plus 20 uM m6BsiRNA1 (No. 6) or plus 40 uM m6BsiRNA1 (No. 7) and monitored for up to 72 hr. Blood from treated mouse was collected within 5 hrs of injection and the serum levels of TNF-α, CCL2, IL-1β and IL-6 was determined by ELISA. As shown in Fig. 5, the injection of LPS alone (positive control) and/or m6BsiRNA2 (negative control) stimulated high levels of serum TNF-α (Fig. 5a, No. 4&5) and CCL2 (Fig. 5b, No. 4&5). However, LPS-induced TNF-α/CCL2 production in the presence of m6BsiRNA1 was reduced by up to 37% (Fig. 5a&b, No. 7) relative to the positive control. Additionally, LPS-induced IL-1β or IL-6 production was not affected in the presence of m6BsiRNA1 (Fig. 5c), suggesting that mSTAT6B does not mediate IL-1β or IL-6 regulation. In order to analyze the effect of m6BsiRNA1 on mSTAT6B expression, protein samples from each experimental group were analyzed by Western blot using antibodies against mSTAT6B, mLITAF and Actin. As shown in Fig. 5a (Western blot, top panel), LPS-induced mSTAT6B expression (No. 4) was significantly reduced in the presence of m6BsiRNA1 (No. 6&7) compared to controls. Altogether, our data show that mSTAT6B may be a important factor that associates with mLITAF to form a complex which translocates into the nucleus. The use of m6BsiRNA1 to inhibit mSTAT6B leads to reduced mLITAF/mSTAT6B-mediated TNF or CCL2 levels in response to LPS.

## Discussion

In this study, we identified the mouse STAT6B gene and characterized its translated protein. Upon LPS stimulation, mLITAF nuclear entry is facilitated by its association with mSTAT6B and the formation of this mSTAT6B/mLITAF complex is crucial for nuclear tranlocation. This translocation leads to TNF and/or CCL2 production as evidenced in our LPS animal model and prevented in LITAF-deficient animals or by using siRNA directed against mSTAT6B.

Based on the present data (See Information S1 and Figure S1), mSTAT6B is highly homologous to human STAT6B [13], but different from human STAT6 [20]. We believe that the C-terminal truncation of mSTAT6B could be resulting from two possible mechanisms: 1) an alternative mRNA splicing product from a single gene and/or 2) proteolytic processing which has only been identified in myeloid cell lines and not in cells of lymphoid lineage [21]. Therefore, we are confident that mSTAT6B is an independent authentic mouse gene. While we were unable to locate this gene in public mouse gene database a possible explanation may be that the gene could be absent from the mouse genome assembly. This is consistent with the recently published information that 1,259 mouse-specific genes were either missing or grossly misassembled in the mouse genome [22]. More accurate annotation may be needed.

Aside from the translocation of the transcription factors inside the nucleus, mSTAT6B functions as an important protein-binding partner to mLITAF in its ability to induce gene transcription and protein expression of CCL2. Although Fig. 3a indicates that mLITAF alone was able to upregulate CCL2 promoter activity, we showed that while mSTAT6B does not directly bind to CCL2 promoter, it is required for an amplified regulation of this promoter by mLITAF. We therefore speculate that the overexpressed mLITAF might interact with endogenous mSTAT6B in the cytoplasm and translocate into the nucleus as a protein complex, where mLITAF acts as a CCL2 regulator with the assistance of mSTAT6B. Furthermore, translocation to nucleus for other transcription factors such as RelA or CSF-1 was not affected by either mLITAF deficiency or mSTAT6B gene knock-down in response to LPS suggesting that 1) treatment of 6BsiRNA1 does not affect RelA or CSF-1 but only mSTAT6B and 2) either mLITAF deficiency or mSTAT6B knock-down results in failure of mLITAF/mSTAT6B protein complex to translocate into the nucleus and mediate the regulation of CCL2 gene expression.

Our data in this study indicates that the critical binding activity was located in the region of CCL2 5′UTR&promoter from −420 to −280. Further analysis of this sequence reveled that CTCCC sequence is found at the repeated region −319∼−315 and −298∼−294. Given that our previous data demonstrated that CTCCC in the region of TNF-alpha promoter is specific to LITAF interaction [23], it is very likely that this sequence is also a regulatory element specific to mLITAF biding activity to other pro-inflammatory cytokines. Additionally, a bioinformatics analysis of promoters for classic mediators of inflammation strongly support the evidence that the presence of CTCCC on their promoter is consistent with a LITAF biological involvement while the lack of CTCCC (IL-6) does not show any LITAF biological involvement.

LPS/TLR2/4/MyD88/p38 MAPK signal transduction generate the formation of the complex mLITAF/mSTAT6B which nuclear translocation leads to the transcription of innate immune response factors such as TNF and CCL2. The knockdown of mSTAT6B by 6BsiRNA1 prevented mLITAF from regulating CCL2 production and tail vein injection of 6BsiRNA1 in mice results in the reduction of LPS-induced serum TNF-α or CCL2 concentration in mice. Furthermore mSTAT6B does not play a role in the regulation of CCL2 production when mLITAF is deficient. Our results highlight the important participation of mLITAF and mSTAT6B in innate immunity suggesting an important role of STAT6B in TNF and CCL2 mediated diseases. The role of m6BsiRNA1 is advocated in potential therapeutics for the treatment of TNF and/or CCL2 diseases.

## Supporting Information

Figure S1 **Identification of** mouse mSTAT6B gene. (a) Diagram of in-frame mSTAT6B cDNA sequence from start to stop codon (1–564 bp). (b) Northern blot. Filter (Clontech) containing preblotted mRNAs (2 µg of each) from different mouse tissues were treated with a α-^32^p-dCTP-labeled mouse mSTAT6B DNA probe (full length size) and an α-^32^p-dCTP-labeled mouse Actin (Amersham Pharmacia, Little Chalfont, UK) as control. The filter was then autoradiographed with Biomax MR film (Kodak). The specific hybrids were indicated by arrows. The assays were done in triplicate and a representative experiment was shown. (c) Mouse chromosomes fixed on the glass slide were hybridized with the biotin-labeled mouse mSTAT6B DNA probe (full-length size). The fluorescent spot, indicated by the arrow, was located on around chromosome 10q10-13. (d) Western blot detection. 100 µg lysate from precultured 1×10^6^ human monocytes (S1, No. 1&3) or mouse primary macrophages (No. 2&4) was detected by western blot with the antibody 5278 (recognition site at N-terminus of mouse mSTAT6B) or STAT6 (recognition site at C-terminus of human STAT6) as shown at bottom. The detected specific protein such as human STAT6 (100 kD), human STAT6B (50 kD) or mouse mSTAT6B (25 kD) was indicated by arrows. The assays were done in triplicate and the representative experiment was shown. (e) Analysis of DNA sequence. According to the analysis for sequence alignment between STAT6 and STAT6B, mouse mSTAT6B (green box) is highly homologous to human STAT6B (light coral box) at N terminus, but DNA translation is stopped at 654 bp downstream of C-terminus of human STAT6B. Mouse mSTAT6B is 89% different from human STAT6 (pink box). The 5278 antibody recognizes the N-terminus of mouse mSTAT6B (indicated by blank arrow), whereas the STAT6 antibody recognizes the C-terminus of human STAT6 (gray arrow).
(TIF)

Figure S2 **Pull-down assay of mLITAF & mSTAT6B.** (a) The primary macrophages collected from LITAF^−/−^ (No. 4&5) or LITAF^+/+^ (No. 1-3) mice were treated with 0.1 µg/ml LPS (Sigma) of either *E. coli* (No. 2&4) or *P. gingivalis* (No. 3&5) or untreated (No. 1) as the control for 2 hrs and washed with PBS and cultured overnight. The cell lysate from each test cell was assessed by Western blot with antibodies against mLITAF, mSTAT6B or Actin as the control. The assays were done in triplicate and a representative experiment was shown. (b) The mouse primary macrophages collected from wild-type mouse (No. 1-6) or from LITAF-deficient genotype mice (No. 7-12) were treated with 0.1 µg/ml *E. coli* LPS (No. 2,4,6,8,10&12) or untreated as the control (No. 1,3,5,7,9&11) for 2 hrs and washed with PBS and cultured overnight. The cell lysate from each treated group was immunoprecipitated (IP) with anti-mLITAF (No. 3,4,9&10) and anti-mSTAT6 (No. 5,6,11&12). The IP-proteins were pulled out with protein A/G beads. The precipitates were subjected to Western blotting with the antibodies against mSTAT6B (5287), mLITAF or Actin as control (indicated by arrows). The assays were done in triplicate and a representative experiment was shown.
(TIF)

Figure S3 **Effect of p38 kinase phosphorylation on LPS-induced mLITAF/mSTAT6B signaling pathway.** (a) The LPS-untreated (No. 1-5) or treated (No. 6-10) mouse primary macrophages were added with various kinase inhibitors (No. 2&7: 5 µM BAY11-7082 for inhibition of NFkB; No. 3&8: 20 µM H-89 for inhibition of Protein kinase A; No. 4&9: 10 µM U0126 for inhibition of Protein kinase or MAK/ERK; No. 5&10: 20 µM SB203580 for inhibition of p38 MAP kinase) and incubated for 3 hrs and washed with PBS. Cells were then cultured overnight. The whole cell lysate or nuclear protein from each test group was harvested separately, purified and subjected to Western blot with antibodies against mLITAF, mSTAT6B (5278) or Actin/tubulin as the control. The assays were done in triplicate and a representative experiment was shown. (b) The LPS-untreated (No. 1-3) or treated (No. 4-6) mouse primary macrophages were added with two kinase inhibitors (No. 2&5: 10 µM U0126 for inhibition of Protein kinase or MAK/ERK; 20 µM SB203580 for inhibition of p38 MAP kinase) and incubated for 3 hrs and washed with PBS. Cells were then cultured overnight. The whole cell lysate from each test group was harvested, purified and subjected to Western blot with antibodies against p-p38, p-NF-kβ p65, pAkt1/2/3 or Actin as the control. The assays were done in triplicate and the representative experiment was shown.
(TIF)

Information S1 (DOC)
