# Supplementary figures and images for: Novel Regulation of CCL2 Gene Expression by Murine LITAF and STAT6B
Source: PLoS One. 2011 Sep 28;6(9):e25083. doi: 10.1371/journal.pone.0025083 (PMC3182193; doi:10.1371/journal.pone.0025083)

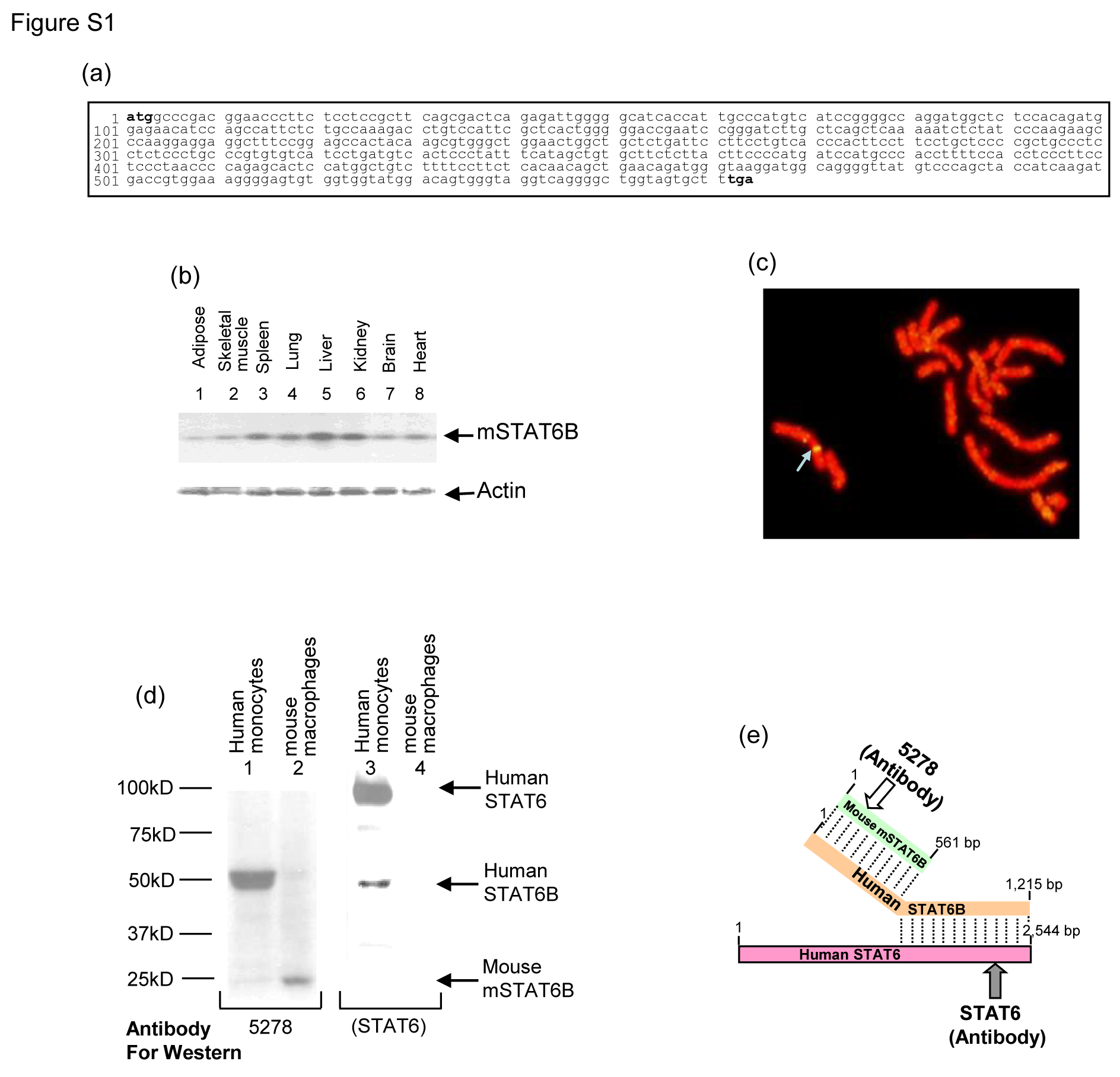

Supplement: Figure S1 — Identification of mouse mSTAT6B gene. (a) Diagram of in-frame mSTAT6B cDNA sequence from start to stop codon (1–564 bp). (b) Northern blot. Filter (Clontech) containing preblotted mRNAs (2 µg of each) from different mouse tissues were treated with a α-32p-dCTP-labeled mouse mSTAT6B DNA probe (full length size) and an α-32p-dCTP-labeled mouse Actin (Amersham Pharmacia, Little Chalfont, UK) as control. The filter was then autoradiographed with Biomax MR film (Kodak). The specific hybrids were indicated by arrows. The assays were done in triplicate and a representative experiment was shown. (c) Mouse chromosomes fixed on the glass slide were hybridized with the biotin-labeled mouse mSTAT6B DNA probe (full-length size). The fluorescent spot, indicated by the arrow, was located on around chromosome 10q10-13. (d) Western blot detection. 100 µg lysate from precultured 1×10^6^ human monocytes (S1, No. 1&3) or mouse primary macrophages (No. 2&4) was detected by western blot with the antibody 5278 (recognition site at N-terminus of mouse mSTAT6B) or STAT6 (recognition site at C-terminus of human STAT6) as shown at bottom. The detected specific protein such as human STAT6 (100 kD), human STAT6B (50 kD) or mouse mSTAT6B (25 kD) was indicated by arrows. The assays were done in triplicate and the representative experiment was shown. (e) Analysis of DNA sequence. According to the analysis for sequence alignment between STAT6 and STAT6B, mouse mSTAT6B (green box) is highly homologous to human STAT6B (light coral box) at N terminus, but DNA translation is stopped at 654 bp downstream of C-terminus of human STAT6B. Mouse mSTAT6B is 89% different from human STAT6 (pink box). The 5278 antibody recognizes the N-terminus of mouse mSTAT6B (indicated by blank arrow), whereas the STAT6 antibody recognizes the C-terminus of human STAT6 (gray arrow). (TIF) [file pone.0025083.s001.tif]

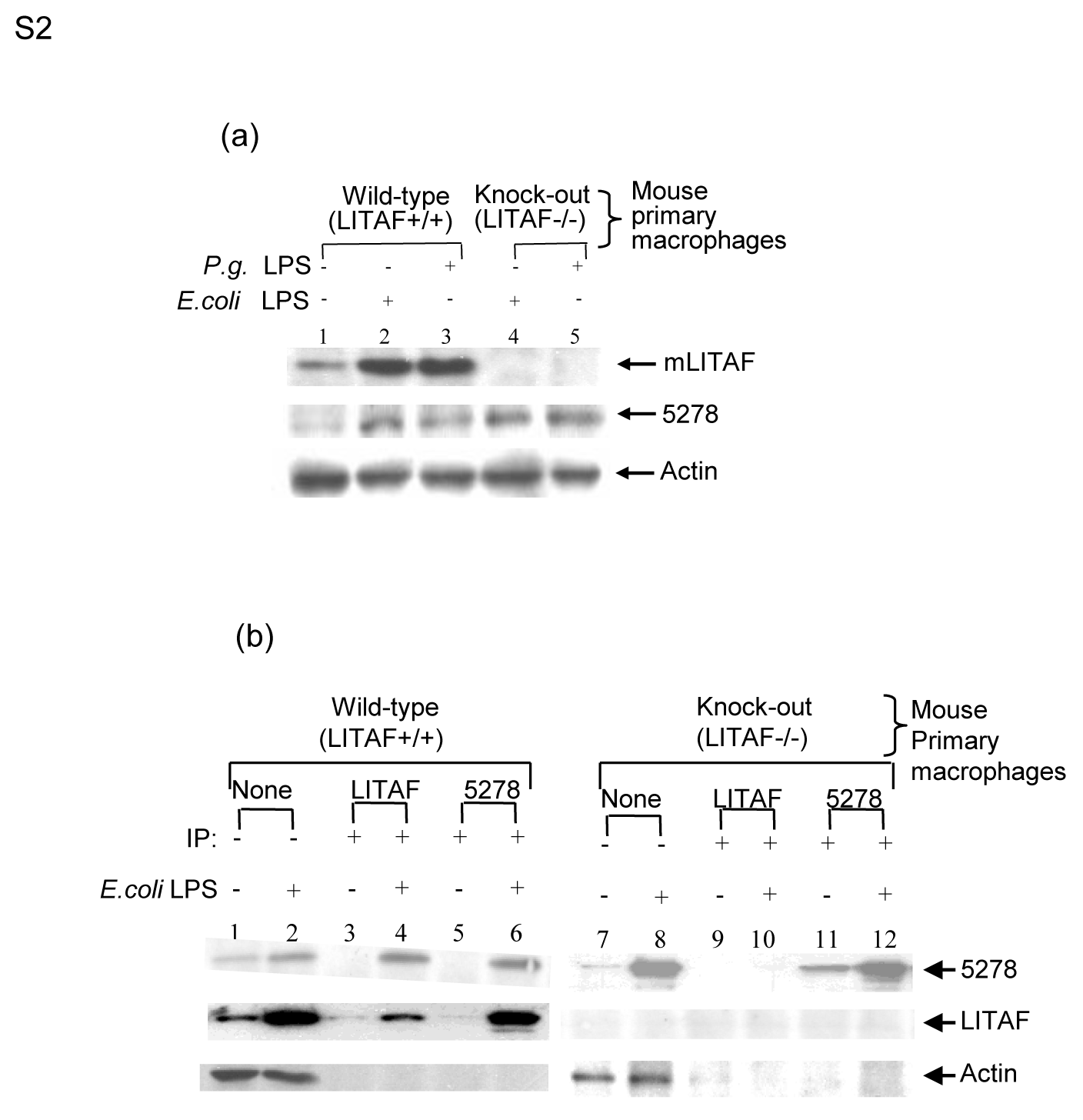

Supplement: Figure S2 — Pull-down assay of mLITAF & mSTAT6B. (a) The primary macrophages collected from LITAF−/− (No. 4&5) or LITAF+/+ (No. 1-3) mice were treated with 0.1 µg/ml LPS (Sigma) of either E. coli (No. 2&4) or P. gingivalis (No. 3&5) or untreated (No. 1) as the control for 2 hrs and washed with PBS and cultured overnight. The cell lysate from each test cell was assessed by Western blot with antibodies against mLITAF, mSTAT6B or Actin as the control. The assays were done in triplicate and a representative experiment was shown. (b) The mouse primary macrophages collected from wild-type mouse (No. 1-6) or from LITAF-deficient genotype mice (No. 7-12) were treated with 0.1 µg/ml E. coli LPS (No. 2,4,6,8,10&12) or untreated as the control (No. 1,3,5,7,9&11) for 2 hrs and washed with PBS and cultured overnight. The cell lysate from each treated group was immunoprecipitated (IP) with anti-mLITAF (No. 3,4,9&10) and anti-mSTAT6 (No. 5,6,11&12). The IP-proteins were pulled out with protein A/G beads. The precipitates were subjected to Western blotting with the antibodies against mSTAT6B (5287), mLITAF or Actin as control (indicated by arrows). The assays were done in triplicate and a representative experiment was shown. (TIF) [file pone.0025083.s002.tif]

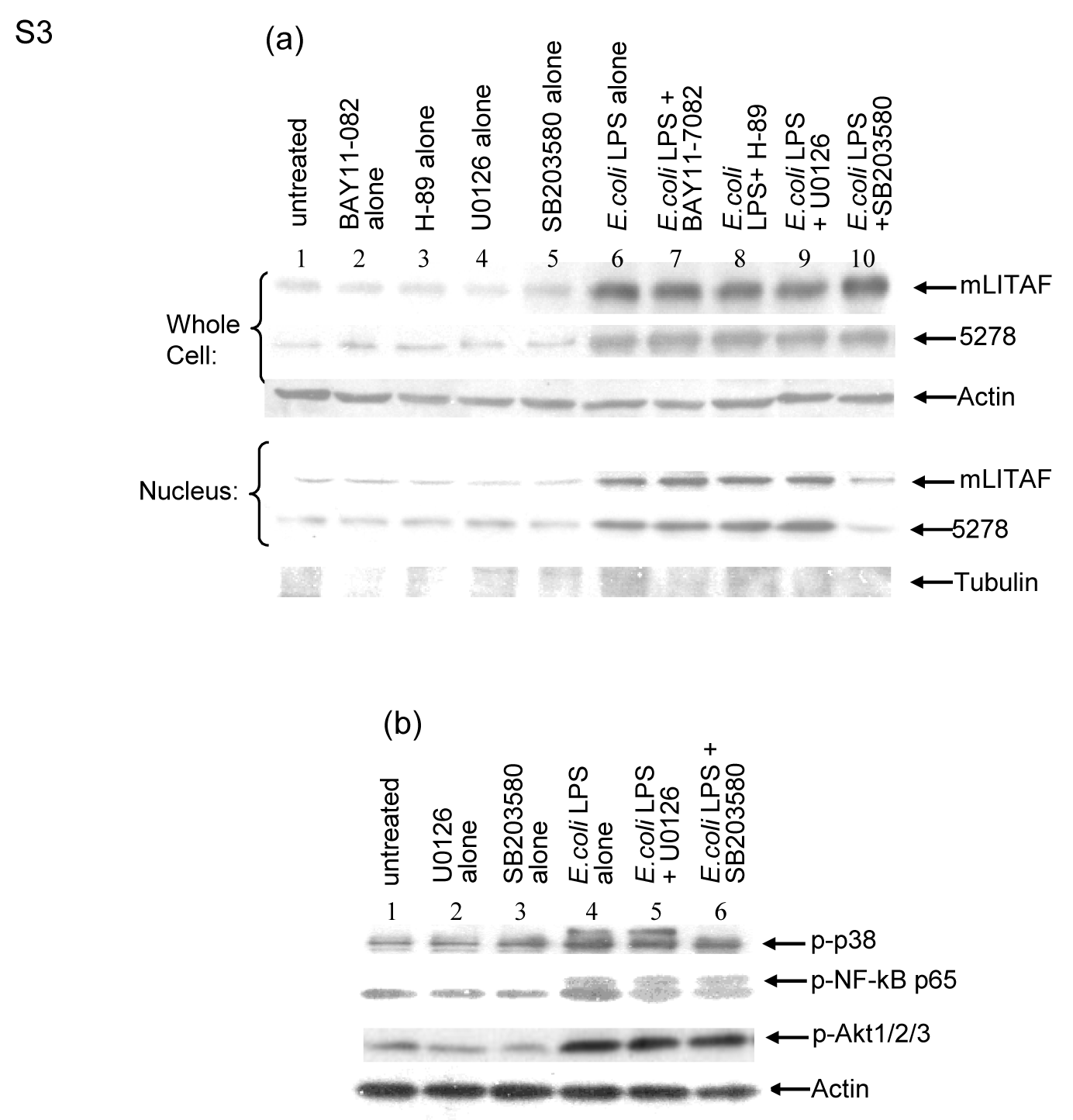

Supplement: Figure S3 — Effect of p38 kinase phosphorylation on LPS-induced mLITAF/mSTAT6B signaling pathway. (a) The LPS-untreated (No. 1-5) or treated (No. 6-10) mouse primary macrophages were added with various kinase inhibitors (No. 2&7: 5 µM BAY11-7082 for inhibition of NFkB; No. 3&8: 20 µM H-89 for inhibition of Protein kinase A; No. 4&9: 10 µM U0126 for inhibition of Protein kinase or MAK/ERK; No. 5&10: 20 µM SB203580 for inhibition of p38 MAP kinase) and incubated for 3 hrs and washed with PBS. Cells were then cultured overnight. The whole cell lysate or nuclear protein from each test group was harvested separately, purified and subjected to Western blot with antibodies against mLITAF, mSTAT6B (5278) or Actin/tubulin as the control. The assays were done in triplicate and a representative experiment was shown. (b) The LPS-untreated (No. 1-3) or treated (No. 4-6) mouse primary macrophages were added with two kinase inhibitors (No. 2&5: 10 µM U0126 for inhibition of Protein kinase or MAK/ERK; 20 µM SB203580 for inhibition of p38 MAP kinase) and incubated for 3 hrs and washed with PBS. Cells were then cultured overnight. The whole cell lysate from each test group was harvested, purified and subjected to Western blot with antibodies against p-p38, p-NF-kβ p65, pAkt1/2/3 or Actin as the control. The assays were done in triplicate and the representative experiment was shown. (TIF) [file pone.0025083.s003.tif]
