# Supplementary material for: Novel Regulation of CCL2 Gene Expression by Murine LITAF and STAT6B
Source: PLoS One. 2011 Sep 28;6(9):e25083. doi: 10.1371/journal.pone.0025083 (PMC3182193; doi:10.1371/journal.pone.0025083)
Supplement: Information S1 — (DOC) [file pone.0025083.s004.doc]

**INFORMATION S1**

**MATERIALS AND METHODS**

**1)Yeast Two-Hybrid Strategy and Screening** Mouse [Liver Matchmaker cDNA Library](http://www.clontech.com/products/detail.asp?tabno=2&catalog_id=638843&page=all) pACT2-cDNAs (Clontech) were used as activation domain (AD) fusion constructs with a selection marker (-Leu) while DNA from pGBKMLF as DNA-BD/bait with a different selection maker (-Trp). Both pACT2-cDNAs (1 μg) and pGBKMLF (1 μg) were cotransformed into AH109-competent yeast cells and cultured in the high-stringency medium with the selection markers SD/-Ade/-His/-Leu/-Trp/X-α-gal, then screened according to the manufacturer’s instruction (Clontech); 2) In situ hybridization. The mouse chromosomes, biotin labeled probe of mSTAT6B (full length size DNA) and the other required materials were prepared. *in situ* hybridization was performed based on the method as described [1]; Northern or Western was performed according to the manufacturer’s instructions. Human monocytes and murine macrophage cells were supplied by ATCC and maintained in DMEM media (Invitrogen, USA) with 10% fetal bovine serum (Amresco, USA) and antibiotics according to standard techniques in cell culture flasks at 37 °C in 5% CO_2_.

Wild-type mice (Jackson Laboratory) and the mLITAF conditional knockout mouse strain (macLITAF^−/−^) was generated by our lab. The primary macrophages were maintained in DMEM medium with 10% FBS and antibiotics in a humidified incubator at 37°C and 5% CO_2_ atmosphere. LPS of *E. coli* 055:B5 was supplied by Sigma (Cat. #L2637). Strain 381 of *P. gingivalis* was grown in brain heart infusion broth with hemin (5 μg/ml) and menadione (1 μg/ml) in an anaerobic atmosphere (85% N_2_/10% H_2_/5% CO_2_) for 24-48 h at 37°C before preparation of LPS as we have described [2].

Kinase Inhibitions, SB203580, U0126, and BAY 11-7082 were purchased from EMD Bioscience, and H-89 from ENZO life Sciences. Mouse macrophages were treated with 20 μM SB203580 (p38 MAPK inhibitor), 10 μM U0126 (ERK or Protein kinase inhibitor), 20μM H-89 (Protein kinase A inhibitor) or 5 μM BAY 11-7082 (NF-kB and IkBα phosphorylation inhibitor) for 3 hrs and washed with PBS. Cells were cultured overnight. The whole cell lysate or nuclear protein from each test was fractionally purified based on the method as described [3].

**RESULTS**

**Characterization of mouse mSTAT6B gene.**

Our previous data indicated that human STAT6B interacts with mLITAF as a transcription factor and mediates TNF-α production in response to LPS stimulation [4]. To determine whether similar functional genes are present in mice, we first employed Yeast Two-Hybrid system for mouse gene screening. mLITAF full-length DNA construct, GBKMLF, as a DNA-BD/bait and Mouse Liver Matchmaker cDNA Library pACT2-cDNAs as AD were cotransformed into yeast AH109 cells. The transformants that contained a specific cDNA surviving in high-stringency medium were screened. Several clones were isolated after high-stringency screenings and the cDNA was confirmed by retransformation and rehybridization with GBKMLF in AH109 cells. One of these clones was a 561-bp cDNA sequence homologous with the human STAT6B (1215bp, Access# AY615283) and was named mSTAT6B (Figure S1a). To further confirm mSTAT6B transcription in mouse, Northern blot analysis was performed using mouse mRNAs from various tissues (Clontech). As shown in Figure S1b, a strong band for mSTAT6B (~1 kb) was identified in mouse spleen, lung, liver, and kidney tissue, and a relatively weak band was detected in brain, heart, skeletal muscle and adipose as well. To genetically characterize mSTAT6B, *in situ* hybridization was performed for designating the chromosomal localization of mSTT6B. Figure S1c shows that the fluorescent spot of mSTAT6B was on chromosome 10q10-13 (indicated by an arrow), which was adjacent to the location of STAT6 on 10q13 [5]. Lastly, Western blot analysis of mSTAT6B protein expression was examined in both human monocytes and mouse primary macrophages. As shown in Figure S1d, by using 5278 antibody, a 25 kD (No. 2) mSTAT6B protein band was detected in mouse macrophages but not in human monocytes (No.1). On the other hand, using STAT6 antibody, production of human STAT6 or human STAT6B was detected in human monocytes (No.3) but no mSTAT6B production was observed in both cells (No.3&4), suggesting that the molecular weight of mouse mSTAT6B is 25 kD and mSTAT6B was only produced in mouse macrophages. As illustrated in Figure S1e, 1-561 bp of mSTAT6B sequence aligned with human STAT6/STAT6B and mSTAT6B was found to end at 561 bp downstream of human STAT6B (green box) and be highly homologous to human STAT6B (light coral box) at N terminus. Additionally, mouse mSTAT6B is 89% different from human STAT6. Taking together, these results provide strong evidence that mSTAT6B and human STAT6/STAT6B are two distinctly different genes.

**LPS-induced mSTAT6B/mLITAF gene expression in mouse primary macrophage cells**.

To determine whether mLITAF) and mSTAT6B function in a cascade in LPS/mLITAF-signaling pathway Western blot was performed. As shown in Figure S2a, mSTAT6B was inducible by LPS in both wild type (No.2&3) and mLITAF knockout (No.4&5) macrophages compared to the control (No.1), which suggested that mSTAT6B was induced independently from mLITAF. We further performed immunoprecipitation to determine whether mLITAF forms a complex with mSTAT6B prior to the translocation. As shown in Figure S2b, the mLITAF-pull-down protein (No.4) was detected by anti-mSTAT6B (5278) antibody, whereas 5278-pull-down protein (No.6) was detected by mLITAF antibody. This suggested that mLITAF associates with mSTAT6B via protein-protein interaction in response to *E. coli* LPS induction.

**Role of p38 MAPK on mLITAF/mSTAT6B protein production in response to LPS.**

Since we showed previously that human LITAF and human STAT6B were phosphorylated by p38 MAP kinase prior to nuclear translocation, and that LPS induced both mLITAF and mSTAT6B, we hypothesized that p38 is involved in a similar way in the mLITAF/mSTAT6B pathway in mouse macrophages. To test this, we treated macrophages with kinase inhibitors including U0126, H-89, Bay11-7082 and SB203580. As shown in Figure S3a, treatment with SB203580 significantly reduced the nuclear translocation of mLITAF/mSTAT6B (No10, lower panel) compared to other treatments (No.6-9). To examine whether SB203580 affects other kinases besides p38, macrophages were treated with either SB203580 or U0126 after stimulation with LPS. According to Figure S3b, SB203580 only inhibited phosphorylated p38 (p-p38, No.6) but not some of other kinases. This suggested that phosphorylation of p38 kinase is involved in LPS/TLR2-4/mLITAF-mSTAT6B signaling pathway.

**REFERENCES**

1. Myokai F, Takashiba S, Lebo R, Amar S (1999) A novel lipopolysaccharide-induced transcription factor regulating tumor necrosis factor alpha gene expression: molecular cloning, sequencing, characterization, and chromosomal assignment. Proc Natl Acad Sci U S A 96: 4518-4523.
2. Zhou Q, Desta T, Fenton M, Graves DT, Amar S (2005) Cytokine profiling of macrophages exposed to Porphyromonas gingivalis, its lipopolysaccharide, or its FimA protein. Infect Immun 73: 935-943.
3. Lindeman GJ, Gaubatz S, Livingston DM., Ginsberg D (1997) The subcellular localization of E2F-4 is cell-cycle dependent. Proc Natl Acad Sci U S A 94: 5095-5100.
4. Tang X, Marciano DL, Leeman SE, Amar S (2005) LPS induces the interaction of a transcription factor, LPS-induced TNF-alpha factor, and STAT6(B) with effects on multiple cytokines. Proc Natl Acad Sci U S A 102: 5132-5137.
5. Patel BK, Keck DL, O’Leary RS, Popescu NC, LaRochelle WJ (1998) Localization of the human stat6 gene to chromosome 12Q13.3-q14.1, a region implicated in multiple solid tumors. Genomics 52: 192-200
